# Olive Nutritional Status and Tolerance to Biotic and Abiotic Stresses

**DOI:** 10.3389/fpls.2019.01151

**Published:** 2019-09-24

**Authors:** Ricardo Fernández-Escobar

**Affiliations:** Departamento de Agronomía, ETSIAM, Universidad de Córdoba, Cordova, Spain

**Keywords:** potassium, calcium, nitrogen, silicon, drought, salinity, temperature, olive leaf spot

## Abstract

The role of nutrients in plant growth is generally explained in terms of their functions in plant metabolism. Nevertheless, there is evidence that plant tolerance or resistance to biotic or abiotic stresses could be affected by the nutritional status. Although not well studied, an adequate nutritional status for optimal plant growth is thought to also be optimal for plant tolerance to stress. Considering the current global trend toward sustainability, studies that clarify the relationships between nutrition and stress are of great interest. For example, potassium plays an important role in the regulation of water status in the olive, improving drought tolerance, while calcium is involved in sodium exclusion mechanism, which can increase tolerance to salinity. Nitrogen excess, in contrast, increases susceptibility to spring frost and olive leaf spot. Silicon is not an essential element for plant growth, but it is considered a beneficial element; among its roles in the control of pests and diseases is the formation of a physical barrier that occurs through silicon deposition in the epidermal cells of the leaves. The presence of soluble silicon also facilitates the deposition of phenolic and other compounds at sites of infection, which is a general defense mechanism to pathogen attack. In olive, silicon application, either by foliar sprays or through irrigation water, reduces the incidence of olive leaf spot. This review summarizes the current status of olive nutrition, the relationships with biotic and abiotic stresses, and the effects of silicon.

## Current Status of Olive Nutrition

The olive, as with other perennial, woody plants, usually requires lower nutrient application than annual plants because it has nutrient storage organs and the ability to reuse these nutrients to support new growth. In mature olive trees growing under rainfed conditions, the amounts of nitrogen, potassium, and calcium removed annually by fruit yield, and pruning were 54.4, 45.5, and 57.9 kg.ha^−1^, respectively (Fernández-Escobar et al., 2015). The amounts of other macronutrients removed, such as phosphorus and magnesium, were less than 7 kg.ha^−1^, while those of micronutrients were less than 0.12 kg.ha^−1^. Under irrigated cultivation, estimations of fruit removal of N, P, and K showed similar or slightly elevated values (Erel et al., 2018). Considering that the current practice in many orchards is to leave a mulch of pruning material, nutrient removal from olive orchards will be lower than the above values.

The small amounts of nutrients removed suggest that, in orchards established on fertile soils, the need for fertilization of olive trees is relatively low. Even with nitrogen, the mineral element required in the greatest amounts by plants, the balance in unfertilized orchards, i.e., the difference between nitrogen input and output that represents the gain or loss of nitrogen in an orchard, was positive (Fernández-Escobar et al., 2012). In contrast, nutrient deficiencies can occur when olive orchards are established on unfertile soils with low availability of a specific nutrient, or when a specific nutrient is blocked due to the physical or chemical characteristics of the soil. Potassium deficiency represents the major nutritional disorder in olives growing both in drylands and on calcareous soils, due to its interaction with water shortage and calcium, respectively (Parra et al., 2003; Restrepo-Díaz et al., 2008). Calcium deficiency is also expected in acidic soils, and deficiencies of other nutrients are occasional and localized. Iron deficiency chlorosis has been reported in calcareous soils in Spain (Fernández-Escobar et al., 1993; Sánchez-Alcalá et al., 2012) and Israel (Zipori et al., 2011). Boron deficiency, meanwhile, has been described in California (Hartmann et al., 1966) and Italy (Sanzani et al., 2012), whereas an excess of boron was observed in some areas of Greece (Chatzissavvidis and Therios, 2010). Zinc deficiency is very rare, although it has been observed in some areas of Turkey (Başar and Gürel, 2016), Sicily (Sanzani et al., 2012), and Israel (Zipori et al., 2018). No references to other nutritional disorders have been reported for olives cultivated under field conditions.

Despite this, large amounts of nutrients are applied annually in many olive orchards. The perception that the annual application of large amounts of fertilizer ensures a good crop, even in orchards established on fertile soils, as well as the low cost of fertilizers in relation to the crop value, has led to unnecessarily high levels of fertilizer application. This practice results in environmental hazards, negative effects on the tree and the crop, and increased costs. **Table 1** shows the negative effects of excess nitrogen application. Nitrogen is the most commonly applied element in olive orchards and is usually applied in excess (Fernández-Escobar, 2011), as also occurs for other fruit tree species (Weinbaum et al., 1992; Faqi et al., 2008). The case of phosphorus is unique. Phosphorus deficiency is very rare in mature fruit trees (Shear and Faust, 1980; Erel et al., 2016), and although there is normally a lack of response to phosphorus application in olive orchards (Hartmann et al., 1966; Jiménez-Moreno and Fernández-Escobar, 2016; Ferreira et al., 2018), phosphorus is still applied in many fertilization programs. However, the reserves of phosphate rock, the main source of phosphorus fertilizer, are finite and could disappear this century (Dawson and Hilton, 2011; Gilbert, 2009), indicating the need for a more responsible and efficient use of phosphorus fertilizers, particularly for woody crops. In contrast, potassium fertilization has been frequently ignored, even though it represents the major nutritional disorder in rainfed olives. This indicates that olive nutrition is based mainly in tradition, repeating the same fertilization program, and also in the testimony of the neighbors. In a study comparing the environmental impacts of several olive-growing systems in Spain, Romero-Gámez et al. (2017) found that the manufacture and application of fertilizers were the primary contributors in all impact categories and cropping systems. These authors concluded that the reduction and optimization of fertilizers would be the most efficient way to improve the sustainability of fertilization practices.

**Table 1:** Negative effects of excess nitrogen on olive trees.

Effect	Reference
Increase soil pollution	Fernández-Escobar et al., 2009a
Decrease oil quality	Fernández-Escobar et al., 2006
	Dag et al., 2018
Reduce flower fertility	Fernández-Escobar et al., 2008
Affects frost tolerance	Fernández-Escobar et al., 2011
Reduce rooting and cutting survival	Dag et al., 2012
Delays fruit maturation	Fernández-Escobar et al., 2014a
Reduce root and shoot growth	Othman and Leskovar, 2019
Reduce nitrogen uptake efficiency	Fernández-Escobar et al., 2014b
Reduce potassium uptake	Antonaya-Baena and Fernández-Escobar, 2012
Increase susceptibility to *Fusicladium oleagineum*	Roca et al., 2018

Performing a rational and sustainable fertilization program is simple. A nutrient should be supplied only when there is evidence that it is needed to assure normal growth and productivity, and when an economic gain is expected from the application of the fertilizer. For this, leaf nutrient analysis is currently the best method available for diagnosing tree nutritional status and the need for fertilization (Shear and Faust, 1980; Benton Jones, 1985; Fernández-Escobar et al., 2009b). Fully expanded leaves from the middle to the basal portion of the current season’s growth must be collected for leaf analysis in July in the northern hemisphere. The analytical results must then be compared with standard values established for these samples, which are shown in **Table 2**. The strategy is to maintain all the nutrients at the adequate levels indicated in the table. When this is achieved, nutrients should not be applied the following season. A nutrient must be applied only if leaf analysis shows values below the sufficiency (adequate) threshold. But if a nutrient is below that threshold because another element interacts with it, usually because it is in excess or deficient, the action should be concentrated on this element. If several nutrients are low (between deficient and adequate) or deficient, application of the lowest or most deficient element is usually enough to correct the problem. If leaf analysis in the following season shows that one of these nutrients is still below sufficiency, it may then be necessary to apply that nutrient. A detailed description of the procedure can be found in Fernández-Escobar (2017) and Fernández-Escobar (2007).

**Table 2:** Interpretation of nutrient levels in olive leaves sampled in July in the northern hemisphere, expressed as dry matter. Adapted from Fernández-Escobar (2018).

Element	Deficient	Adequate	Toxic
Nitrogen, N (%)^1^	1.4 (1.2)	1.5-2.0 (1.3-1.7)	(>1.7)
Phosphorus, P (%)^2^	0.05	0.1-0.3	–
Potassium, K (%)	0.4	>0.8	–
Calcium, Ca (%)	0.3	>1	–
Magnesium, Mg (%)	0.08	>0.1	–
Manganese, Mn (ppm)	–	>20	–
Zinc, Zn (ppm)	–	>10	–
Copper, Cu (ppm)	–	>4	–
Boron, B (ppm)	14	19-150	185
Sodium, Na (%)	–	–	>0.2
Chloride, Cl (%)	–	–	>0.5

^1^In brackets, nitrogen levels proposed by Molina-Soria and Fernández-Escobar (2012).

^2^Toxicity symptoms were observed at 0.21% in young plants (Jiménez-Moreno and Fernández-Escobar, 2016).

Estimating the amount of fertilizer required when leaf analysis indicates the need for fertilization is not simple. It should be based on judgments of tree nutritional status, crop demand, nutrient availability, and other site-specific variables. Nutrient removal of a deficient element is a simple first approach to determine the amount of that nutrient to be applied. Additionally, Fernández-Escobar (2017) suggests application rates that can be used to correct deficiencies.

The timing of nutrient application must consider that the uptake and use of a nutrient are usually not simultaneous processes. Thus, the aim of fertilization is to maximize nutrient uptake efficiency to increase its content in the tree, which normally occurs when the tree is active. Once taken up, the nutrient may be stored in the tree or used for growth.

## Relationships With Biotic and Abiotic Stresses

The roles of nutrients in plant growth can usually be explained in terms of their functions in plant metabolism. However, there is evidence that plant tolerance or resistance to biotic or abiotic stresses can be affected by the nutritional status (Huber et al., 2012; Sanzani et al., 2012). These relationships have not been well studied, particularly in woody plants, but it is generally assumed that an adequate nutritional status for optimal plant growth is also optimal for plant tolerance to these stresses. Consequently, good management of olive nutrition, as described above, may mitigate the negative effects of some biotic or abiotic stresses.

Some abiotic factors are well known to influence tolerance to pests and disease (Sanzani et al., 2012). In contrast, it is still unclear how global warming affects the incidence of current olive pests or pathogens, or the emergence of new ones. Nevertheless, all practices in olive culture should encourage sustainability, and studies that clarify the relationships between olive nutrition and biotic and abiotic stresses will be of great interest, particularly considering that European regulations have led to a drastic reduction in the number of authorized active materials for olive pest and disease control.

### The Role of Mineral Nutrients in the Tolerance to Abiotic Stresses

The olive is a crop that is commonly subjected to various stress situations (Sanzani et al., 2012). It is traditionally cultivated in marginal areas, often on calcareous soils, and usually under rainfed conditions, but is sometimes also irrigated with low-quality water. Although cultivated almost exclusively under Mediterranean conditions, olive orchards can be found in many different regions with diverse climates. Many older olive orchards are still based on traditional growing systems, but new plantations are based on more intensive growing methods. The use of new varieties, different cultural practices, and new environments may influence the effects of abiotic stresses.

Since most olive orchards are cultivated in drylands, K deficiencies can be expected (Restrepo-Díaz et al., 2008). There is insufficient information available to fully understand the role of nutritional status on drought tolerance. However, it is well known that K-deficient plants lose water more easily than well-nourished ones (Hsiao and Läuchli, 1986; Fournier et al., 2005). One plausible explanation may be that K plays an important role in stomatal opening and closure (Poffenroth et al., 1992) and, consequently, in the regulation of plant water status. In the olive, stomatal conductance under drought conditions is reportedly higher in K-deficient plants than in well-nourished ones displaying lower water-use efficiency (Arquero et al., 2006; Benlloch-González et al., 2008). Erel et al. (2014) reported that, under these conditions, the addition of Na^+^ increases stomatal conductance. Since K plays an important role in the regulation of water status in olive trees, maintaining leaf K concentrations above the sufficiency threshold is highly recommended. However, it has also been reported that K uptake by olive trees is restricted by both K deficiency of the tree and water stress (Restrepo-Díaz et al., 2008), indicating that K fertilizer must be applied before the deficiency threshold is reached, and when trees present a good water status, usually in spring if growing in drylands.

Water demand for irrigation is increasing in olive orchards because of increased yields. Since olive trees are considered moderately tolerant to salinity (Maas and Hoffman, 1977; Rugini and Fedeli, 1990; Marin et al., 1995) and water resources in the Mediterranean basin are scarce, irrigation with saline water is often used. As in other glycophytic species (Greenway and Munns, 1980), salt tolerance in the olive is associated with ion exclusion mechanisms located in the roots (Benlloch et al., 1991; Tattini et al., 1995; Melgar et al., 2006), which reduce translocation and accumulation of Na^+^ and Cl^−^ to the aerial part. Olive trees are less sensitive to leaf Cl^−^ than Na^+^ (Bongi and Loreto, 1989; Tattini et al., 1992), and the Cl^−^ ion does not cause toxicity in olive trees (Melgar et al., 2009). In contrast, Na^+^ toxicity is usually a concern when Ca^2+^ concentrations are relatively low (Bañuls et al., 1991; Maas, 1993). Indeed, when Ca^2+^ was applied to saline irrigation water, leaf Ca^2+^ concentrations increased with the amount of Ca^2+^ applied, but with a concomitant decrease in leaf Na^+^ concentrations (Melgar et al., 2006). This indicates that Ca^2+^ plays an important role in the Na^+^ exclusion mechanism, and that maintaining adequate Ca^2+^ nutrition can protect olive trees against Na^+^ toxicity (Melgar et al., 2007; Tattini and Traversi, 2009; Methenni et al., 2018). The results obtained from a long-term experiment under field conditions (Melgar et al., 2009) suggest that supplying Ca^2+^ in irrigation water to prevent Na^+^ toxicity using drip irrigation until winter rest, as well as growing a tolerant cultivar, can allow the use of high-saline irrigation water for extended periods without affecting olive tree growth or yield.

The olive is more tolerant to high than low temperatures (Sebastiani, 2018). Damage due to high temperatures is very rare in the olive and is often associated with drought or dry, hot winds (Sanzani et al., 2012). Under these conditions, Benlloch-González et al. (2016) found that the olive tree has a great capacity to accumulate K in the root, which is used by the tree to maintain the growth of the aerial part since, as mentioned previously, K plays an important role in the regulation of water status in the olive. However, when high temperatures affect both the aerial part and root system, both K transport from the roots and tree growth are inhibited.

Low temperatures in winter and spring are limiting for olive growth. According to Sanzani et al. (2012), the extent of frost damage depends on the time of the year when the frost occurs. Little damage is expected during the fall, but in winter, the aerial part of the plant can be damaged at temperatures below −7°C (Palliotti and Bongi, 1996), and tree death can occur at −12°C (Larcher, 1970). In spring, olive trees are susceptible to frost injury. The threshold temperature below which symptoms of cold damage appear depends on numerous factors, including the nutritional status of the tree. Nitrogen has been associated with frost tolerance in many crops, although the study results are controversial. Depending on the species and/or the reference, increasing plant N may increase, decrease, or have no influence on cold tolerance (Pellett and Carter, 1981). In a study developed to determine the influence of N status on the frost tolerance of olive trees under field conditions, Fernández-Escobar et al. (2011) found that, in October, before the onset of dormancy, excess N resulted in increased frost tolerance. During dormancy, all the trees exhibited greater tolerance to low temperatures, and no differences were observed among them. In April, after budbreak, trees become more sensitive, and those with an excess of N were more sensitive to low temperatures. These results may explain some of the controversial results reported in the literature for other species. In Italy, K fertilization is recommended to reduce frost damage in the spring (Proietti and Famiani, 2005), likely due to the role of K in the regulation of water status.

Interactions among mineral nutrients are frequent, particularly in the soil. For instance, it is well known that K, Ca, and Mg interact in the soil exchange complex, sometimes inducing deficiencies in one element through an excess of another. Interactions also occur at the plant level, such that a deficiency or excess of one element may affect the uptake or utilization of another. However, the exact nature of some of these interactions remains unclear. As mentioned above, the main nutritional concerns in many olive orchards growing in the Mediterranean area, particularly in Spain, are low or deficient levels of plant K as well as N overfertilization. Reports regarding N and K interactions are scarce and mostly refer to annual plants. In the olive, preliminary results obtained with potted plants showed a significant interaction between N and K. Plants well-nourished with K increased both K content and vegetative growth, depending on the amount of N applied. In contrast, plants poorly nourished with K exhibited decreased K content and vegetative growth at the highest amounts of N applied (Antonaya-Baena and Fernández-Escobar, 2012). These results indicate that K uptake could be reduced in K-deficient plants with an excess of N. Moreover, leaf K concentrations are low in many Spanish olive orchards growing in soils with high levels of available K but subjected to N overfertilization.

### The Role of Mineral Nutrients in the Tolerance to Biotic Stresses

Although plant tolerance to pests and disease is a genetic characteristic, it can also be affected by environmental factors like plant nutrition. The effect depends on the nutrient, plant species, and parasite, although it may be small in very tolerant or very susceptible cultivars.

Many studies have been carried out on the effects of N and K, and their interaction, on plant tolerance to pests and disease (Huber et al., 2012), although the results have sometimes been controversial. However, overall, excess N tends to favor disease development (Huber and Thompson, 2007), while K deficiency increases susceptibility to parasites (Huber and Arny, 1985). The efficiency of disease control also depends on the N:K ratio, since the effect of K may vary depending on the plant N nutritional status (Prabhu et al., 2007). The role of Ca is also well documented, particularly its effects on disease control during fruit storage (Shear, 1975; Scott and Wills, 1979; Rahman and Punja, 2007). Other mineral nutrients can also affect disease incidence, but to a lesser extent (Datnoff et al., 2007; Marchner, 2012), although Cu is used extensively as a fungicide for many species, including the olive. In addition, the role of phosphite [insert inline here] in the control of *Phytophthora cinnamomi* in many tree species is well documented (Fenn and Coffey, 1984; Fernández-Escobar et al., 1999).

Reports relating to olive nutrition and tolerance to biotic stresses are limited. Recent studies showed the effect of N on the incidence of olive leaf spot, the most common foliar disease in the olive, which is caused by the fungus *Fusicladium oleagineum* (currently *Venturia oleaginea*). Experiments developed under different growing conditions, including hydroponic culture, potted plants, and mature trees growing under field conditions, showed that disease incidence was significantly higher in plants subjected to a high-N treatment than in those subjected to a low-N treatment under all conditions (Roca et al., 2018). These results suggest that N excess increases susceptibility to olive leaf spot.

Mycorrhization of nursery olive plants has become common practice as it results in increased plant tolerance to biotic and abiotic stresses (Castillo et al., 2006; Porras-Soriano et al., 2009). However, mycorrhization of young olive plants with *Glomus intraradices* (currently *Rhizophagus irregularis*) has recently been observed to interact with the presence of high levels of P in the substrate (Jiménez-Moreno et al., 2018), resulting in reduced plant growth. In contrast, when the substrate contained low P concentrations, root growth increased compared to a control without mycorrhization. These results suggest that P should not be supplied to the substrate if plants are to be mycorrhized.

Tree tolerance to pest attack often depends more on the presence of repellents or toxic compounds than the effect of a nutrient (Huber et al., 2012). However, young plants or those with rapid vegetative growth are generally more susceptible to pest attack. In the olive, excessive N fertilizer application may increase the incidence of *Saissetia oleae*, likely due to the emergence of numerous new shoots that facilitates a suitable place for nymphs to settle (Ben-Dov and Hodgson, 1997).

### The Role of Non-Essential Elements: Silicon

Some elements are not essential for plant growth, but are considered to have beneficial effects on plants, particularly due to their roles in plant tolerance to biotic and abiotic stresses. One such element is silicon (Si), the second most abundant element in the earth’s crust after oxygen. Silicon in soils can be found in a solid phase composed mainly of silica (SiO_2_) and silicates adsorbed to soil particles and Fe and Al oxides and hydroxides, or in a liquid phase mainly in the form of monosilicic acid (H_4_SiO_4_) (Tubana et al., 2016). Monosilicic acid does not dissociate at pHs below 9 and plants uptake Si from the soil solution in this form (Epstein, 1994; Ma and Takahashi, 2002). All plants growing in soil contain Si in their tissues, and the concentration of Si in plant tissues depends on the soil and plant species (Tubana et al., 2016; Debona et al., 2017).

The Si taken up by plants is transported from the roots to shoots, either actively or passively, *via* the transpiration stream. In the shoot, silicic acid is concentrated through loss of water due to transpiration and is polymerized into insoluble silica, forming a silica gel layer between the cuticle and epidermal cells (Debona et al., 2017; Wang et al., 2017) and preventing Si translocation to new, growing leaves. This silica layer constitutes a physical barrier that is believed to reduce the incidence of pests and disease, as well as to improve photosynthetic rates and tolerance to water stress, drought conditions, and other abiotic stresses (Marchner, 2012). Silicon also forms a chemical barrier, inducing the production of phenolic compounds, phytoalexins, and other products that activate plant defense mechanisms (Debona et al., 2017; Wang et al., 2017).

Consequently, plants supplied with Si may have enhanced resistance to multiple biotic and abiotic stresses (Luyckx et al., 2017). Ma (2004) suggests that the more Si accumulates in the shoots, the larger the effect. Therefore, although Si is abundant in the soil, many plants cannot take up enough Si to increase their tolerance to biotic and abiotic stresses. Since Si deposited in the leaves is immobile, a continuous supply of Si is required in newly formed leaves for optimal tolerance to these stresses (Huber et al., 2012). Silicon can be applied by foliar sprays, directly to the soil, or through irrigation water, although Savva and Ntatsi (2015) indicated that application through the soil is more effective than foliar application in increasing Si levels in plant tissues.

Although many studies investigating the effect of Si application in plants have recently been undertaken, limited information is available on Si application in fruit tree crops. In the olive, recent studies reported that continuous Si application in young olive plants significantly reduces symptoms in leaves inoculated with *V. oleaginea*, even at low doses (Nascimento-Silva et al., 2018). Cultivar differences were evident in the responses to Si application, and the effect was more pronounced in 'Arbequina', a moderately susceptible cultivar, than in the more susceptible cultivar 'Picual'. Additionally, this study clearly showed that both foliar spray and application through irrigation water were effective in increasing leaf Si concentrations. However, at the highest doses applied, foliar sprays were more effective at increasing leaf Si concentrations in 'Arbequina', but not 'Picual'. These results are interesting because most olive trees are grown in drylands, and foliar spraying is the method commonly used for agrochemical applications.

## Author Contributions

The author confirms being the sole contributor of this work and has approved it for publication.

## Funding

This work was supported by project AGL2017-85246-R financed by the Agencia Estatal de Investigación and European Regional Development Funds (AEI/FEDER, UE).

## Conflict of Interest Statement

The author declares that the research was conducted in the absence of any commercial or financial relationships that could be construed as a potential conflict of interest.
